# Emotional support networks and discussion of anal cancer among sexual minority men in Nigeria: insights from an ego-centric analysis

**DOI:** 10.1186/s12889-026-27674-w

**Published:** 2026-05-30

**Authors:** Connor R. Volpi, John Chama, Ruxton Adebiyi, Jumoke A. Aigoro, Yerima Jibrin Bawa, Kazeem E. Kolawole, Uchenna Ononaku, Naomi Ayuba, Ashley Shutt, Michelle B. Shin, Abayomi Aka, Stephen E. Goldstone, Patrick Dakum, Joel M. Palefsky, Sylvia Adebajo, Rebecca G. Nowak, Karin E. Tobin

**Affiliations:** 1 Johns Hopkins Bloomberg School of Public Health, Baltimore, MD USA; 2 Division of Epidemiology and Prevention, Institute of Human Virology, University of Maryland School of Medicine, 725 W. Lombard Street, Baltimore, MD 21201 USA; 3 Institute of Human Virology Nigeria, Abuja, Nigeria; 4 University of Washington, Seattle, WA USA; 5 International Centre for Advocacy on Rights to Health, Abuja, Nigeria; 6 Icahn School of Medicine at Mount Sinai, New York, NY USA; 7 University of California, San Francisco, San Francisco, CA USA

**Keywords:** Sexual minority men (SMM), HIV, Emotional support networks, Anal cancer prevention, HPV vaccination, Nigeria

## Abstract

**Background:**

This study examined the role of emotional support networks in shaping health-related communication among sexual minority men (SMM) living with HIV in Nigeria, a context where criminalization and stigma limit social integration and access to care. We conducted a cross-sectional survey of 250 SMM receiving HIV care at an SMM-affirming clinic in Abuja.

**Methods:**

Participants completed a structured questionnaire assessing demographics, HIV-related characteristics, HPV knowledge, anal cancer awareness, and emotional support network composition. Emotional support network attributes were compared by duration of HIV diagnosis (≤ 5 years vs. >5 years), and logistic regression models assessed factors associated with discussing anal cancer within support networks.

**Results:**

Participants living with HIV for > 5 years had larger emotional support networks and were more likely to report discussing same-sex relationship issues and anal cancer with alters. Discussing same-sex relationships and receiving medical care support from alters were strongly associated with increased likelihood of discussing anal cancer. Network density (e.g., how well alters know one another) did not differ by duration of HIV diagnosis.

**Conclusion:**

These findings highlight the influence of interpersonal trust and relationship context on communication about stigmatized health topics. Leveraging emotional support networks, particularly for individuals newly diagnosed with HIV, may strengthen engagement in preventive cancer care and improve health outcomes for SMM in hostile sociopolitical environments.

## Introduction

Social support networks play a critical role in the well-being of sexual minority men (SMM) living with HIV, influencing health behaviors, emotional resilience and access to care [1–3]. In Nigeria, where homosexuality is criminalized [4], SMM face unique social and structural barriers that may impact their ability to form and maintain supportive networks [5–7]. Prior research has demonstrated that strong social support is associated with improved antiretroviral therapy (ART) adherence [8], reduced psychological distress [9] and greater engagement in preventive health measures among SMM living with HIV [10]. However, the composition and characteristics of these networks may change over time, with potential differences in emotional support and overall network structure based on the duration of HIV diagnosis [11, 12].

In social network research, it is useful to distinguish between a person’s total network, which includes all individuals with whom they interact [13], and their emotional support network, which specifically comprises those who provide meaningful psychological and emotional support [14]. These networks are often analyzed using an ego-centric approach [15], where the individual (the “ego”) and their direct ties (“alters”) are the focus, allowing for closer examination of the support structures immediately surrounding the person. Guided by social network theory, we conceptualize emotional support networks as relational contexts that shape health communication through mechanisms including trust, disclosure safety, shared identity, and perceived norms [16]. Within these networks, individuals may feel greater psychological safety to discuss stigmatized health concerns, particularly when alters share similar lived experiences or identities [17].

The emotional support network is particularly important for mental health and coping among SMM living with HIV [18], especially in environments where stigma and legal restrictions constrain broader social integration [19]. However, despite its relevance, the structure and dynamics of emotional support networks among SMM remain understudied. The size, density, and composition of these networks may shift over time as individuals adapt to their HIV diagnosis, form and maintain relationships, and engage with supportive healthcare environments [20–22], including SMM-affirming clinics that provide critical support services.

In addition to the psychosocial challenges faced by SMM living with HIV in Nigeria, they are also at heightened risk for anal cancer due to persistent high-risk human papillomavirus (HPV) infection [23]. In addition to high-risk HPV infection, smoking is an established co-factor in anal carcinogenesis, potentially through impaired immune response, increased HPV persistence, and promotion of oncogenic progression [24]. As smoking prevalence remains high among SMM living with HIV, it represents an important behavioral risk factor to consider in studies of anal cancer prevention [25]. Despite elevated risk, awareness and discussion of anal cancer prevention, including HPV vaccination and anal cancer screening, remain limited among SMM in Nigeria [26], and cost further constrains access even when services are available [27]. Stigma [28], criminalization [29] and lack of provider awareness [30] contribute to the underutilization of preventive services, which are themselves scarce and largely concentrated in a few urban centers [31].

Given this context, emotional support social networks may play a crucial role in facilitating discussions about anal cancer prevention and shaping intentions to engage in preventive health behaviors, even when service availability is limited. Their influence may operate through several mechanisms. First, emotional support networks may provide disclosure safety [17], allowing individuals to discuss stigmatized topics such as anal health without fear of judgement or legal repercussions. Second, shared identity within networks of other SMM may normalize conversations about HPV vaccination and screening [32]. Third, trusted alters may serve as credible messengers, increasing confidence in prevention information and reducing uncertainty about navigating services [33]. These mechanisms may be particularly salient among those accessing care at SMM-affirming clinics, where openness and trust can strengthen peer support and information sharing [34–36]. In this framework, emotional support network characteristics are conceptualized as upstream social determinants of anal cancer communication [37, 38], which may influence intentions and uptake of preventive behaviors.

This study examined the demographic and behavioral characteristics of SMM living with HIV and their emotional support networks, stratified by the duration of HIV diagnosis (≤ 5 years vs. >5 years). The five-year cutoff was selected to distinguish earlier versus more established post-diagnosis adaptation and approximated the median duration of HIV diagnosis in the sample. Prior work has shown that time since diagnosis shapes how individuals adapt socially and mobilize support [39, 40], which in turn influence network structure and engagement in preventive health behaviors. We aimed to investigate how emotional support networks influence health-related discussions and engagement in preventive care among SMM in Nigeria which is complex given the heightened vulnerability related to both HIV and legal persecution [41]. By comparing the characteristics of alters—social network members—by duration of HIV diagnosis, we aimed to identify key factors that shape how SMM engage in discussions about health and relationships within their support networks.

## Methods

### Study design and population

This cross-sectional study was conducted as part of a broader anal cancer prevention initiative at an SMM-affirming clinic (TRUST) in Abuja, Nigeria [42]. Between April 1 and June 14, 2024, 250 SMM living with HIV were recruited to participate in a survey assessing HPV knowledge, anal cancer symptom awareness and HPV vaccine acceptability. A community partner of TRUST, the International Center for Advocacy on Right to Health (ICARH) assisted with recruitment. Eligible participants were required to be 18 years or older, self-identify as a SMM, and receive HIV care at the participating clinic. The sample, therefore, reflects clinic-engaged SMM and may differ systematically from SMM not engaged in care. Written informed consent was obtained from all participants prior to enrollment. The study received ethical approval from the National Health Research Ethics Committee in Nigeria (NHREC/01/01/2007- 18/01/2024), the University of Maryland, Baltimore (HP-00108629), and Johns Hopkins University (IRB00027747) and covered data collection between January 12th -September 6th, 2024.

### Data collection

Data were collected through a structured electronic questionnaire administered via REDCap [43], available in both English and Hausa. The survey gathered participant information (e.g., age, smoking status, duration of HIV diagnosis), assessed HPV knowledge using binary yes/no questions, and measured anal cancer symptom awareness. Age relative to ego was included in multivariable models to account for generational differences within support networks. Participant chronological age was not included because it was highly correlated with duration of HIV diagnosis. Furthermore, smoking status was included given its established role in HPV persistence and anal carcinogenesis and was treated as a potential confounder in analyses.

HPV vaccine acceptability was evaluated on a 0–10 Likert scale (0 = strongly disagree; 10 = strongly agree); after participants were informed that the vaccine can prevent both anal cancer and anogenital warts. Survey content was informed by prior instruments used in HPV and cancer prevention research among key populations, as well as constructs from health behavior frameworks emphasizing knowledge and perceived acceptability in shaping preventive uptake. Respondents were asked to provide emotional support network information for up to 5 people. To ensure cultural relevance and comprehension, the questionnaire was developed in collaboration with local healthcare providers through iterative feedback sessions and cognitive interviewing [44]. The questionnaire was then piloted with a subset of SMM receiving care at the clinic to refine wording, structure and clarity prior to full implementation.

### Statistical analyses

Descriptive statistics were used to summarize the demographic, behavioral and emotional support network characteristics of study participants. Continuous variables were reported as means with standard deviations (SD) or medians with interquartile ranges (IQR), as appropriate, while categorical variables were presented as frequencies and percentages.

Comparisons between groups were conducted using chi-square tests for categorical variables and t-tests or Wilcoxon rank-sum tests for continuous variables, depending on distribution. In descriptive analyses, time since HIV diagnosis (≤ 5 years vs. >5 years) was the grouping variable. Social network characteristics were the primary variables of interest, and demographic and behavioral characteristics were compared across groups to describe differences between participants. No covariate adjustment was performed in these bivariate comparisons.

Logistic regression models were used to examine correlates of anal cancer discussions; additional models examined HPV knowledge and anal cancer symptom awareness. In the primary multivariable logistic regression, the outcome was whether the participant reported any anal cancer discussion discussing. The model included duration since HIV diagnosis (≤ 5 years vs. >5 years), age relative to ego (older/same/younger), sexual identity, how alters were met, ability to discuss same-sex relationship issues with alters, and medical care support. Anal cancer discussion was operationalized as any self-reported conversation with a named emotional support network member about anal cancer, including prevention (e.g., HPV vaccination), screening (e.g., anal cancer screening or high-resolution anoscopy), symptoms, risk factors, or treatment. Chronological age was not included in the primary multivariable model because of collinearity with duration since HIV diagnosis.

All analyses were conducted at the participant (ego) level, with one observation per ego. Alter-level characteristics were summarized to the ego level. Because the analytic dataset includes one row per participant, observations were independent across participants, and no clustering adjustment was required. Covariates were selected based on prior literature and theoretical relevance to anal cancer communication and social networks. Risk behaviors were assessed descriptively through self-reported items on the number of male sexual partners in the past month and engagement in condomless anal sex during the same period. Participants were asked whether they had discussed specific health topics with each named alter (e.g., individuals in their immediate, ego-centric network), enabling assessment of health-related communication within their emotional support networks.

Emotional support network characteristics were assessed using name generator items that captured network density (e.g., how well alters know one another), relationship strength (e.g., perceived closeness) and support provision (e.g., emotional and informational support). Network density was calculated as the proportion of observed connections among alters out of all possible connections. Higher-density networks were hypothesized to facilitate anal cancer discussions because interconnected alters may reinforce shared norms, increase accountability, and enhance diffusion of health information within the network. Conversely, lower-density networks may provide greater privacy and reduce concerns about information spreading beyond trusted ties. Given these competing mechanisms, density was examined to assess whether network cohesion or privacy was more salient for anal cancer communication.

Medical care support was defined as assistance provided by an alter related to healthcare engagement, including encouragement to seek care, accompaniment to clinic visits, assistance navigating services, or emotional support related to medical decision-making. Additional analyses examined whether participants discussed relationship issues (same-sex and opposite-sex partners) with alters and described alter characteristics (e.g., sexual identity). All statistical analyses were conducted in Stata [45], with statistical significance defined as *p* < 0.05.

## Results

### Participant characteristics by duration of HIV diagnosis

Table 1 summarizes demographic and behavioral characteristics of participants by years living with HIV (≤ 5 vs. >5 years). The mean age was 30 years, with those living with HIV > 5 years significantly older (35 vs. 27 years, *p* < 0.001). Education did not differ by group (*p* = 0.31), but unemployment was more common in the ≤ 5-year group (72.9% vs. 54.7%, *p* = 0.004). Income reporting also varied, as refusal was higher among those with > 5 years of HIV (58.5% vs. 29.2%, *p* = 0.002). Marital status differed between groups, with more participants in the > 5-year group married (34.0% vs. 9.0%, *p* < 0.001).

**Table 1:** Demographic characteristics of participant (“ego”) by years living with HIV

	Total (*N* = 250)	≤5 years (*N* = 144)	> 5 years (*N* = 106)	*p*-value*
Age, Mean (SD):	30.12 (6.22)	26.66 (4.42)	34.84 (5.11)	**< 0.001**
Highest level of education, N (%)				
Junior Secondary/JSS Senior Secondary/SSS Higher than SSS	51 (20.4) 110 (44.0) 89 (35.6)	20 (13.9) 88 (61.1) 36 (25.0)	31 (29.3) 22 (20.9) 53 (50.0)	0.31
Currently employed, N (%)				
No Yes Refusal	163 (65.2) 86 (34.4) 1 (0.4)	105 (72.9) 39 (27.1) 0 (0.0)	58 (54.7) 47 (44.3) 1 (0.9)	**0.004**
Monthly income (Naira), N (%)				
< 70,000 ≥70,000 Refusal	108 (43.2) 104 (41.6) 38 (15.2)	21 (14.6) 81 (56.3) 42 (29.2)	17 (16.0) 27 (25.5) 62 (58.5)	**0.002**
Marital status, N (%)				
Married Not Married	49 (19.6) 201 (80.4)	13 (9.0) 131 (91.0)	36 (34.0) 70 (66.0)	**< 0.001**
Number of male partners in the last month, Mean (SD):				0.55
	6.03 (4.59)	5.88 (4.49)	6.24 (4.7)	
Anal sex without a condom in the past one month, N (%)				0.12
No Yes Refusal	105 (42.0) 142 (56.8) 3 (1.2)	66 (45.8) 75 (52.1) 3 (2.1)	39 (36.8) 67 (63.2) 0 (0.0)	
Current tobacco use, N (%)				
No Yes Refusal	121 (48.4) 128 (51.2) 1 (0.4)	85 (59.0) 58 (40.3) 1 (0.7)	36 (34.0) 70 (66.0) 0 (0.0)	**< 0.001**
Current party drug use, N (%)				
No Yes Refusal	157 (62.8) 90 (36.0) 3 (1.2)	99 (68.8) 43 (29.9) 2 (1.4)	58 (54.7) 47 (44.3) 1 (0.9)	**0.02**
Number of alters in the social network, Mean (SD):				**0.03**
	3.04 (1.21)	2.90 (1.15)	3.24 (1.25)	
Density of alters in the social network, Mean (SD):				0.44
	0.50 (0.50)	0.48 (0.44)	0.53 (0.46)	

*p-values were calculated using two-sample t-tests for continuous variables and chi-square tests for categorical variables

Bold indicates statistical significance (*p* <0.05)

*Abbreviations*: *SD* Standard deviation

Risk behaviors such as number of male partners and condomless anal sex showed no differences (*p* = 0.55, *p* = 0.12), but tobacco use (66.0% vs. 40.3%, *p* < 0.001) and party drug use (44.3% vs. 29.9%, *p* = 0.02) were significantly higher in the > 5-year group. Emotional support network size was also larger among those with > 5 years of HIV (3.24 vs. 2.90 alters, *p* = 0.03), but network density did not differ (*p* = 0.44).

### Alter characteristics by duration of HIV diagnosis

Table 2 summarizes alter characteristics by years living with HIV (≤ 5 vs. >5 years). Alters (e.g., the social network members named by each participant, or ego) with > 5 years of HIV were younger (38.5% vs. 23.0% under younger age categories, *p* < 0.001), while education, occupation and marital status showed no differences. Relationship strength was slightly higher in the ≤ 5-year group (Mean = 6.04 vs. 5.74, *p* = 0.02). Alters of those with > 5 years were more likely to discuss same-sex relationship issues (46.7% vs. 34.3%, *p* < 0.001), though no differences were seen for opposite-sex relationships (*p* = 0.55) or sexual partnership status (*p* = 0.47). Friend alters were more common in the > 5-year group (*p* = 0.03).

**Table 2:** Characteristics of alters by years living with HIV

	Total Alters Named	≤5 years (*N*=417)	>5 years (*N*=343)	*p*-value*
Age relative to ego				<0.001
Older	196 (25.8)	129 (30.9)	67 (19.5)	
Same age	335 (44.1)	191 (45.8)	144 (42.0)	
Younger	228 (30.0)	96 (23.0)	132 (38.5)	
Don’t know	1 (0.1)	1 (0.2)	0 (0.0)	
Level of education				0.83
≤Junior Secondary/JSS	35 (4.6)	19 (4.6)	16 (4.7)	
Senior Secondary/SSS	175 (23.0)	97 (23.3)	78 (22.7)	
Higher than SSS	421 (55.4)	235 (56.4)	186 (54.2)	
Don’t know	129 (17.0)	66 (15.8)	63 (18.4)	
Occupation				0.15
Working	376 (49.5)	208 (49.9)	168 (49.0)	
Not Working	30 (4.0)	18 (4.3)	12 (3.5)	
Student	133 (17.5)	83 (19.9)	50 (14.6)	
Other	170 (22.4)	82 (19.7)	88 (25.7)	
Don’t know	51 (6.7)	51 (6.7)	25 (7.2)	
Marital status				0.47
Married	113 (14.9)	56 (13.4)	57 (16.6)	
Not Married	634 (83.4)	354 (84.9)	280 (81.6)	
Don’t know	13 (1.7)	7 (1.7)	6 (1.8)	
Strength of relationship, Mean (SD)				**0.02**
	5.90 (1.9)	6.04 (1.9)	5.74 (1.7)	
Able to discuss same-sex partner relationship issues				**<0.001**
No	448 (59.0)	272 (65.2)	176 (51.3)	
Yes	303 (39.9)	143 (34.3)	160 (46.7)	
Don’t know	6 (0.8)	2 (0.5)	4 (1.2)	
Refusal	3 (0.4)	0 (0.0)	3 (0.9)	
Able to discuss opposite-sex partner relationship issues				0.55
No	383 (50.4)	205 (49.2)	178 (51.9)	
Yes	374 (49.2)	211 (50.6)	163 (47.5)	
Don’t know	3 (0.4)	1 (0.3)	2 (0.6)	
Sexual partner?				0.47
No	173 (22.8)	90 (21.6)	83 (24.2)	
Yes	586 (77.1)	326 (78.2)	260 (75.8)	
Refusal	1 (0.1)	1 (0.2)	0 (0.0)	
Relation				**0.03**
Relative	35 (20.2)	24 (26.7)	11 (13.3)	
Friend	138 (79.8)	66 (73.3)	72 (86.8)	
Total^†^	173 (100.0)	90 (100.0)	83 (100.0)	
How did you meet?				**0.01**
Family member	38 (5.0)	28 (6.7)	10 (2.9)	
Through other friends	418 (55.0)	231 (55.4)	187 (54.5)	
Internet/social media	144 (19.0)	88 (21.1)	56 (16.3)	
TRUST/ICARH	89 (11.7)	42 (10.1)	47 (13.7)	
Another OSS	65 (8.6)	27 (6.5)	38 (11.1)	
Don’t know	1 (0.1)	1 (0.3)	0 (0.0)	
Refusal	5 (0.7)	0 (0.0)	5 (1.5)	
Sexual identity				**<0.001**
Gay or homosexual	102 (13.4)	69 (16.6)	33 (9.6)	
Bisexual, queer, or other	608 (80.0)	315 (75.5)	293 (85.4)	
Heterosexual or straight	44 (5.8)	32 (7.7)	12 (3.5)	
Don’t know	6 (0.8)	1 (0.3)	5 (1.5)	
Sex at birth				0.13
Female	34 (4.5)	23 (5.5)	11 (3.2)	
Male	726 (95.5)	394 (94.5)	332 (96.8)	
Able to show love and affection?				0.12
No	246 (32.4)	128 (30.7)	118 (34.4)	
Yes	510 (67.1)	285 (68.4)	225 (65.6)	
Don’t know	4 (0.5)	4 (1.0)	0 (0.0)	
Provide emotional support?				0.2
No	269 (35.4)	139 (33.3)	130 (37.9)	
Yes	489 (64.3)	276 (66.2)	213 (62.1)	
Don’t know	2 (0.3)	2 (0.5)	0 (0.0)	
Able to show love and affection?				0.37
No	648 (85.3)	352 (84.4)	296 (86.3)	
Yes	110 (14.5)	63 (15.1)	47 (13.7)	
Don’t know	2 (0.3)	2 (0.5)	0 (0.0)	
Living with HIV?				0.5
No	303 (39.9)	167 (40.1)	136 (39.7)	
Yes	183 (24.1)	94 (22.5)	89 (26.0)	
Don’t know	274 (36.1)	156 (37.4)	118 (34.4)	
Aware of HIV status of ego?				0.69
No	531 (69.9)	290 (69.5)	241 (70.3)	
Yes	166 (21.8)	91 (21.8)	75 (21.9)	
Don’t know	62 (8.2)	36 (8.6)	26 (7.6)	
Refusal	1 (0.1)	0 (0.0)	1 (0.3)	
Encourages ART adherence:				0.13
No	32 (19.3)	21 (23.1)	11 (14.7)	
Yes	132 (79.5)	70 (76.9)	62 (82.7)	
Don’t know	2 (1.2)	0 (0.0)	2 (2.7)	
Total^‡^	166 (100.0)	91 (100.0)	75 (100.0)	
Help with medical care?				**0.05**
No	533 (70.1)	287 (68.8)	246 (71.7)	
Yes	216 (28.4)	120 (28.8)	96 (28.0)	
Don’t know	11 (1.5)	10 (2.4)	1 (0.3)	
Talked about health issues before?				0.15
No	185 (24.3)	113 (27.1)	72 (21.0)	
Yes	573 (75.4)	303 (72.7)	270 (78.7)	
Don’t know	2 (0.3)	1 (0.3)	1 (0.3)	
Discussed topics related to anal cancer?				**<0.001**
No	353 (46.5)	220 (52.8)	133 (38.8)	
Yes	404 (53.2)	194 (46.5)	210 (61.2)	
Don’t know	3 (0.4)	3 (0.7)	0 (0.0)	
Comfortable talking about topics related to anal cancer:				**0.01**
No	321 (42.2)	192 (46.0)	129 (37.6)	
Yes	420 (55.3)	218 (52.3)	202 (58.9)	
Don’t know	4 (0.5)	4 (1.0)	0 (0.0)	
Refusal	15 (2.0)	3 (0.7)	12 (3.5)	
Comfortable encouraging alters to engage in anal cancer screening?				**0.05**
No	320 (42.1)	188 (45.1)	132 (38.5)	
Yes	423 (55.7)	218 (52.3)	205 (59.8)	
Don’t know	5 (0.7)	5 (1.2)	0 (0.0)	
Refusal	12 (1.6)	6 (1.4)	6 (1.8)	
Encourages condom use:				0.05
No	344 (45.3)	177 (42.5)	167 (48.7)	
Yes	410 (54.0)	239 (57.3)	171 (49.9)	
Don’t know	3 (0.4)	1 (0.2)	2 (0.6)	
Refusal	3 (0.4)	0 (0.0)	3 (0.9)	
Encourages smoking cessation:				0.64
No	286 (73.5)	124 (74.7)	162 (72.7)	
Yes	102 (26.2)	42 (25.3)	60 (26.9)	
Don’t know	1 (0.3)	0 (0.0)	1 (0.5)	
Total^§^	389 (100.0)	166 (100.0)	223 (100.0)	

*p-values were calculated using chi-square tests for categorical variables and Wilcoxon rank-sum tests for continuous variables

^†^Relation was assessed only among alters not identified as sexual partners (*n* = 173)

^‡^Encourages ART adherence was assessed only among alters aware of the participant’s HIV status (*n* = 166)

^§^Encourages smoking cessation was assessed only among alters of participants who reported current smoking; totals reflect this subset (*n* = 389)

Bold indicates statistical significance (*p* <0.05)

Alter sexual identity also varied, with more bisexual/queer alters among the > 5-year group (85.4% vs. 75.5%, *p* < 0.001). Sex at birth distributions did not differ (*p* = 0.13). Emotional support indicators—advice, affection, and general support—showed no significant group differences (*p* > 0.05). By contrast, discussions of anal cancer were significantly more common among alters of the > 5-year group (61.2% vs. 46.5%, *p* < 0.001), who also reported greater comfort (*p* = 0.01) and more frequent encouragement to undergo screening (*p* = 0.05). Condom use encouragement was marginally higher (*p* = 0.05), but ART adherence (*p* = 0.13) and smoking cessation support (*p* = 0.64) did not differ.

## Factors associated with anal cancer discussions

Table 3 shows factors associated with participants’ ability to discuss anal cancer from logistic regression analyses. In unadjusted models, having younger alters was associated with lower odds of discussing anal cancer (OR = 0.59, 95% CI: 0.35–0.98, *p* = 0.043), but this association was not significant after adjustment (aOR = 0.80, 95% CI: 0.42–1.51, *p* = 0.49). Compared with older alters, same age alters were not associated with discussing anal cancer in adjusted analyses (aOR = 0.89; 95% CI: 0.48–1.64; *p* = 0.70). Duration of HIV diagnosis (> 5 years vs. ≤5 years) was not independently associated with discussing anal cancer after adjustment (aOR = 1.41; 95% CI: 0.77–2.60; *p* = 0.266).

**Table 3:** Bivariable and multivariable logistic analyses of factors associated with ego’s ability to discuss anal cancer (*n* = 250)

Variable	OR (95% CI)	aOR (95% CI)
Years living with HIV		
≤5 years > 5 years	(ref.) 1.18 (0.77–2.60)	(ref.) 1.41 (0.77–2.60)
Age relative to ego		
Older Same age Younger	(ref.) 1.04 (0.62–1.76) **0.59 (0.35–0.98)**	(ref.) 0.89 (0.48–1.64) 0.80 (0.42–1.51)
Able to discuss same-sex partner relationship issues		
No Yes	(ref.) **1.53 (1.24–1.90)**	(ref.) **2.13 (1.18–3.85)**
Sexual identity		
Heterosexual or straight Gay or homosexual Bisexual, queer, or other	(ref.) 1.48 (0.73–2.95) 1.15 (0.56–2.36)	
How did you meet?		
Family member Through other friends Internet/social media TRUST/ICARH Another OSS	(ref.) 1.66 (0.89–3.11) 0.63 (0.37–1.06) 1.60 (0.83–3.07) 1.30 (0.65–2.58)	
Help with medical care?		
No Yes	(ref.) **3.44 (2.27–5.20)**	(ref.) **8.09 (4.22–15.50)**

*Abbreviations*:* aOR* Adjusted odds ratio, *CI* Confidence interval, *ref.* Reference group

Bold indicates statistical significance (*p* <0.05)

Being able to discuss same-sex relationship issues with alters remained significantly associated with discussing anal cancer (aOR = 2.13, 95% CI: 1.18–3.85, *p* = 0.012). Where egos met their alters showed mixed, nonsignificant trends: meeting through friends increased odds (aOR = 1.66, 95% CI: 0.89–3.11), while meeting via social media decreased odds (aOR = 0.63, 95% CI: 0.37–1.06). Meeting through community organizations (TRUST/ICARH or other SMM-affirming clinics]) also showed no significant associations. The strongest predictor was receiving medical care support from alters, which was associated with nearly ninefold higher odds in adjusted analyses (aOR = 8.94; 95% CI: 4.81–16.61, *p* < 0.001).

## Discussion

This study highlights the potential role of emotional support networks in shaping cancer-related communication and engagement in preventive health discussions among SMM living with HIV in Nigeria [1–3]. Given the criminalization of homosexuality in the country [4], SMM face structural and social barriers that may hinder their ability to form and maintain strong support systems [5–7]. Participants who had been living with HIV for more than five years exhibited larger emotional support networks. These differences may reflect age, life-course stage, relationship stability, or post-diagnosis adaptation processes [11, 12]. These individuals were also more likely to engage in discussions about same-sex relationship issues and anal cancer-related topics compared to those with a shorter duration of HIV diagnosis. The observed association between discussions of same-sex relationship issues and anal cancer conversations suggests that interpersonal dynamics may influence health communication. Fostering stigma-free environments [46], such as those facilitated by the TRUST clinic, could help support more open dialogue around cancer prevention among SMM.

Prior research has shown that SMM living with HIV are at increased risk of anal cancer due to persistent high-risk HPV infections. [23, 47] However, stigma, lack of provider awareness, and structural barriers to care may shape how egos and alters discuss, or avoid discussing, prevention, screening, and HPV vaccination [28–30], potentially limiting the flow of health-promoting information within support networks. Our results indicate that having alters who provide medical care support was strongly associated with discussing anal cancer, consistent with the idea that trusted social connections may support engagement with preventive healthcare services. This strong association between receiving medical care support from alters and discussing anal cancer highlights the value of integrating trusted support persons into health education and intervention strategies to promote cancer-related communication and engagement in preventive care among SMM.

Network size and relationship strength varied across participants and were examined as potential correlates of health-related discussions; however, network density did not differ significantly by duration of HIV diagnosis. The lack of association between network density and anal cancer discussion suggests that overall network interconnectedness may be less influential than specific supportive relationships. Although individuals living with HIV for more than five years had larger support networks, the overall interconnectedness of those networks remained similar across groups, suggesting relatively stable patterns of network cohesion over time. The observed association between marital status and years living with HIV may reflect increasing relationship stability, which could influence long-term engagement in care and access to emotional or practical support [48, 49].

Moreover, discussing same-sex relationship issues within social networks was strongly associated with increased likelihood of discussing anal cancer, emphasizing the interconnection between relationship dynamics and health communication. Our finding that alters met through mutual friends were associated with higher odds of discussing anal cancer, whereas those met through the internet or social media were associated with lower odds, suggests that the context in which relationships are formed influences comfort with sensitive health discussions. [50] While many SMM in Nigeria do meet partners and peers online due to stigma and safety concerns [51], these ties may initially be less trusted or less intimate than relationships formed through existing social circles, which could limit willingness to discuss health topics.

Given these findings, our results extend prior work showing the importance of leveraging emotional support networks to improve health literacy and preventive care uptake [20–22]. This may be especially critical for individuals newly diagnosed with HIV, who may not yet have well-established support systems. Strategies such as peer-led education, community engagement programs, and provider training on culturally competent care can enhance awareness of anal cancer prevention and increase uptake of screening services [41].

These findings have important implications for HIV clinics serving SMM in stigmatizing contexts. Clinics that provide affirming and culturally competent care may serve not only as sites of clinical service delivery but also as hubs that strengthen supportive social ties and foster open dialogue about stigmatized health conditions [52, 53]. Integrating structured opportunities for peer interaction within HIV clinics, such as support groups or facilitated discussions, may enhance the circulation of cancer prevention information within emotional support networks [54, 55]. Routine incorporation of anal cancer education, HPV vaccination counseling, and screening discussions into HIV care visits may also normalize these topics and reinforce communication beyond the clinic setting.

Our findings further support the potential of peer navigator and network-based interventions to promote anal cancer communication and preventive behaviors. Individuals who receive medical care support from trusted alters were significantly more likely to report discussing anal cancer, suggesting that leveraging respected peers within emotional support networks may increase awareness and uptake of prevention services. Network-informed strategies, such as identifying influential peers, training peer navigators to disseminate HPV-related information, and equipping trusted network members with culturally appropriate education tools, may be particularly effective in contexts where stigma limits formal health communication channels [56]. Such approaches may be especially impactful for individuals newly diagnosed with HIV who are still developing stable support networks [57].

This study provides valuable insights into the role of social support networks in shaping health behaviors among SMM living with HIV in a highly stigmatized environment. By focusing on individuals engaged in care at an SMM-affirming clinic, it captures unique social dynamics that influence discussions on preventive health, including anal cancer prevention. The use of electronic data collection enhanced accuracy and confidentiality and incorporating both demographic and social network characteristics allowed for a nuanced analysis of health-related discussions. However, we were unable to determine the motivations underlying alters’ support for prevention (e.g., health benefit vs. financial incentive), and this remains an area for future investigation.

However, the cross-sectional design limits the ability to determine causal relationships between social networks and health behaviors. Self-reported data may introduce recall and social desirability biases, particularly regarding sensitive health topics. We also did not assess internalized homophobia or related stigma constructs, which prior research suggests may influence both social connectedness and engagement in preventive health behaviors [58]. Internalized stigma may shape willingness to disclose sexual identity, discuss anal cancer, or seek screening and vaccination services, and may mediate or moderate associations between emotional support networks and health communication [59, 60]. Future research should explicitly incorporate measures of internalized stigma to better understand these multilevel dynamics.

Age relative to ego was included to capture generational dynamics within support networks; participant chronological age was not included because it was strongly correlated with duration since HIV diagnosis. Observed differences by duration since HIV diagnosis may partially reflect life course factors rather than time since diagnosis alone. Prior research suggests that willingness to disclose health concerns, engage peer support, and navigate care systems evolves across the life span [61]. Future longitudinal research is needed to disentangle age-related effects from duration of HIV diagnosis. Additionally, findings may not be generalizable to SMM who are not engaged in affirming care or who face greater barriers to accessing healthcare. There is also the possibility that multiple egos named the same alter, which may lead to overlap in network data and affect interpretations of network influence.

## Conclusion

This study underscores the role of emotional support networks in shaping discussions about stigmatized topics such as anal cancer and same-sex relationships among SMM living with HIV in Nigeria. In a context of criminalization and persistent stigma, these networks may provide important contexts for discussing health concerns and navigating preventive care. Our findings suggest that longer duration of HIV diagnosis was associated with larger emotional support networks; however, this relationship may also reflect age-related or life-course factors rather than duration since diagnosis alone.

These results highlight how cancer-related communication circulates within support networks and point to the potential of SMM-affirming healthcare and peer support to strengthen HPV-related cancer prevention. Future research should examine longitudinal changes in support networks and identify strategies to harness close interpersonal relationships for improving cancer prevention among SMM living with HIV.

## Data Availability

The data used for this study is available upon reasonable request from the corresponding author.

## Abbreviations

aOR: Adjusted Odds Ratio
ART: Antiretroviral Therapy
CI: Confidence Interval
HIV: Human Immunodeficiency Virus
HPV: Human Papillomavirus
ICARH: International Centre for Advocacy on Right to Health
IQR: Interquartile Range
IRB: Institutional Review Board
NHREC: National Health Research Ethic Committee
OR: Odds Ratio
SD: Standard Deviation
SMM: Sexual Minority Men
